# Determination of detection depth of optical probe in pedicle screw measurement device

**DOI:** 10.1186/1475-925X-13-148

**Published:** 2014-11-01

**Authors:** Weitao Li, Yangyang Liu, Zhiyu Qian

**Affiliations:** Department of Biomedical Engineering, Nanjing University of Aeronautics and Astronautics, Yudao Street, Nanjing, China

**Keywords:** Detection depth, Pedicle screw, Near infrared spectra, Navigation

## Abstract

**Background:**

There is a high probability of accidental perforation of the vertebral pedicle wall in pedicle screw insertion surgery. A pedicle screw (PS) measurement device with an optical probe has been reported to send out a warning signal before the PS tip breaking the vertebral pedicle wall.

**Methods:**

In this study, we explored the detection depth of optical probe in this measurement device, which was closely related to the effective alarm distance. In the boundary, the vertebrae tissues could be treated as 2-layer models including spongy bones and compact bones. The Monte Carlo simulation and phantom models were performed to analyse and define the detection depth. Then the porcine vertebrae models were performed to obtain optical spectrum and reduced scattering coefficient, based on which the detection depths were deduced. Moreover, a comparison was made to explore the most significant pattern factor from the experiment results.

**Results:**

According to the pattern factor, an alarm threshold was successfully deduced to define the alarm distance during pedicle screw monitoring.

**Conclusions:**

Thus, the proposed alarm standard based on detection depth provides a potential for guiding pedicle screw in surgery.

## Background

Pedicle screw (PS) fixation has been widely used as a clinical fusion operation. Several methods have been reported to increase the accuracy of pedicle screw placement [1, 2], such as somatosensory evoked potentials (SSEP), motor evoked potentials, compound muscle action potentials (CMAP), electromyography recordings (EMG) [3, 4], computed tomography (CT) [5, 6], intra-operative fluoroscopy [7, 8] and computer-aided frameless stereo axis [9–11]. However, there is still a high probability of accidental perforation of the vertebral pedicle wall in PS insertion surgery.

Considering the limitations of the current guidance aids, the technique based on near-infrared spectrum (NIRs) was designed to guide the PS during pedicle surgery [12, 13]. In the former study, we have developed a measurement device, which could differentiate vertebrae tissues in PS placement by using an optical fiber probe and analyzing diffuse reflectance spectra [14–16]. The optical fiber probe used in the monitoring device contains one source optical fiber and one detection optical fiber with a core-to-core separation of 200 μm. This optical fiber probe enables us to obtain one-dimensional spatial mapping profiles along the PS tracks in real time. Moreover, this optical fiber probe has been used to investigate and identify optical properties of biological tissue, diagnose cancer, and monitor drug delivery process [17–19]. Recently, some reports have demonstrated that this optical fiber probe could reliably detect millimetre layers of intracranial white matter structures, based on rodent models, intralipid (IL) phantoms, and Monte Carlo studies [20, 21].

When the tissue is irradiated by light, multiple elastic scattering and absorption will happen inside the tissue. So the reflectance spectra can provide information on scattered size and densities within the measured tissue, which are also influenced by the parameters of optical fiber probe and light source [22–24]. Meanwhile, because of the scattering nature of light, valuable physiological information provided by reflectance spectra is poor in the spatial accuracy [25, 26]. According to the structure of optical fiber probe, only the properties of the tissue in front of the probe tip can be detected and deduced from the reflectance spectra. The maximum distance of the interrogated volume ahead of the probe highly depends on the optical properties of the measured sample and the structure of the source and detection optical fibers. Here, we defined detection depth being from the tip of the probe to the maximum detection distance, which comes from a concept of “lookthrough distance” [27]. It can be calculated from the reflectance spectra [28].

In this study, the experiments and simulations were performed to obtain the detection depth of such optical fiber probe in order to identify and investigate an important judgment standard to alarm accidental perforation, when this probe was used in PS insertion surgery. First, the Monte Carlo simulation was presented to address the detection depth of the NIRs small-separation probe by using obtained parameters of different tissues (*e.g.* reduced scattering coefficient (*μ*^*′*^_*s*_) and absorption coefficient (*μ*_*a*_)). Second, intralipid phantom was prepared to simulate the pedicle bones. Third, the pedicle screw monitoring device with a needle-like optical fiber probe was employed to explore the real detection depth in vertebrae bones, and different optical parameters were analysed and compared. Finally, alarm threshold was obtained and applied to alarm cortical breach during pedicle screw monitoring.

## Methods

### Dual-fiber reflectance experimental setup

**Figure 1:**
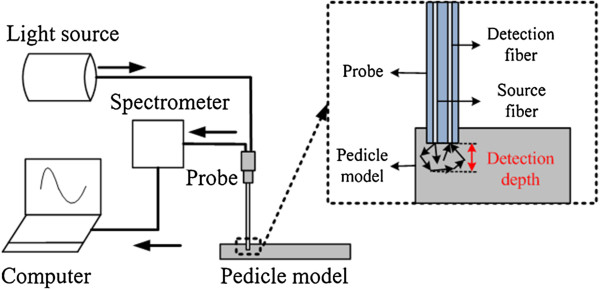
**NIRs measurement device.**

The dual-fiber NIRs measurement device used in this paper is described in Figure 1, which consists of a stabilized fan-cooled broadband light source (HL 2000, Ocean Optics Inc.), a spectrometer (USB2000, Ocean Optics Inc.), a needle-like dual-fiber probe that is connected to the light source and the spectrometer. Two 200 μm-diameter fibers are arranged in parallel at a core-to-core distance of 200 μm in a steel tube. Light is delivered by one optical fiber to the surface of tissue, which is shown in Figure 1 (dotted arrow). Detection depth represents the distance between the tip of the probe and the maximum forward depth in the pedicle bone models.

Pattern factors of different tissues in vertebra bones are deduced (*e.g. μ*^*′*^_*s*_ and *μ*_*a*_). The calculation details of *μ*^*′*^_*s*_ and *μ*_*a*_ have been described previously [29]. Therefore, the *μ*^*′*^_*s*_ (at 690 nm) and the *μ*_*a*_ (at 690 nm) can be measured by this NIRs measurement device. In addition, the results of bones have been validated by Oximeter (model 96208, ISS Inc.) [30].

### Monte Carlo simulations of detection depth

**Figure 2:**
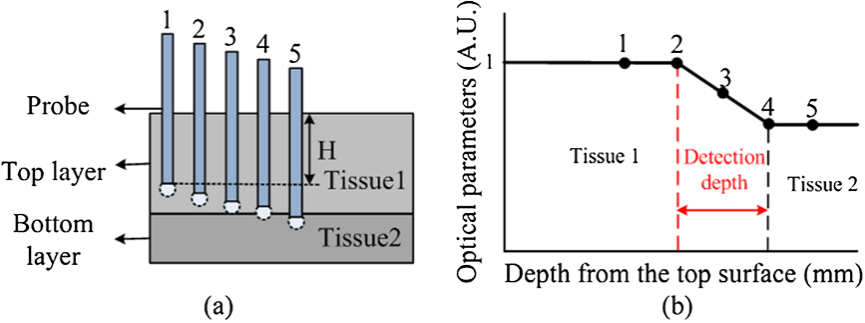
**Monte Carlo simulation. (a)** Monte Carlo simulation model **(b)** hypothesis about detection depth. H represents the depth from the top surface.

Monte Carlo simulation was employed to predict the optical reflectance from the vertebra and the detection depth crossing a boundary, which was provided by Wang and Jacques [31, 32]. Here, we hypothesize that the optical properties in different bones tissues are distinguished. A two-layer model is designed (Figure 2) to verify this hypothesis. In Figure 2(a), tissue 1 (top layer) and tissue 2 (bottom layer) are two different tissues in vertebral bones. When the probe is located at point 1 to 5, the optical properties curve is given in Figure 2(b). Then the detection depth will be deduced. H is used as abscissa in the next simulation and experiments, which is the depth from the top surface. Point 1,2,3,4 and 5 represent the different locations of the probe. The solid black line in Figure 2(b) is the hypothesis curve.

In this simulation, the number of photons was 1,000,000, and the indexes of refraction (*n*) for both top and bottom layers were set to 1.38 [33] since both of them were simulating vertebra tissues. The mean cosine of the scattering angle (*g*) for tissue was 0.9. The simulated core-to-core separation between the source and detector was 200 μm, and the output data were normalized at a certain depth of the 2-layer model. Some other parameters are shown in Table 1. Here, the probe was placed within the tissue volume for both the phantom and the animal studies. The simulation parameters were chosen in such a way that there existed no mismatch of *n* during the entry process. Specifically, we used 1.38 for the top entry medium so that there was no air-tissue interface (or mismatch of *n*) when the injected photons entered the top layer. Furthermore, the reflectance spectra were acquired when the probe was moving from the surface of the top layer to the bottom layer. The thickness of tissue 1 was 5 mm and tissue 2 was 2 mm, shown in Figure 2(a). The step size was 0.2 mm when the probe was 0–4 mm away from the top surface, and was 0.1 mm when the probe was crossing the boundary between the two layers (4 mm to 6 mm away from the top surface). Two-layer models were used in the simulation to represent compact bone and spongy bone with specified optical parameters of *μ*^*′*^_*s*_ and *μ*_*a*_ at 690 nm. Therefore, the top layer has *μ*^*′*^_*s*_ = 17.23 cm^-1^ and *μ*_*a*_ = 0.41 cm^-1^, and the bottom layer has *μ*^*′*^_*s*_ = 12.02 cm^-1^ and *μ*_*a*_ = 0.21 cm^-1^.

**Table 1:** **Monte Carlo simulation parameters**

Photon injection	Detection angles	Voxel size	Boundary conditions
An infinitely narrow collimated beam	Vertical to the surface (90°)	Axial: 0.02 cm Lateral: 0.005 cm	Dirichlet boundary condition

### Intralipid phantom measurements

**Figure 3:**
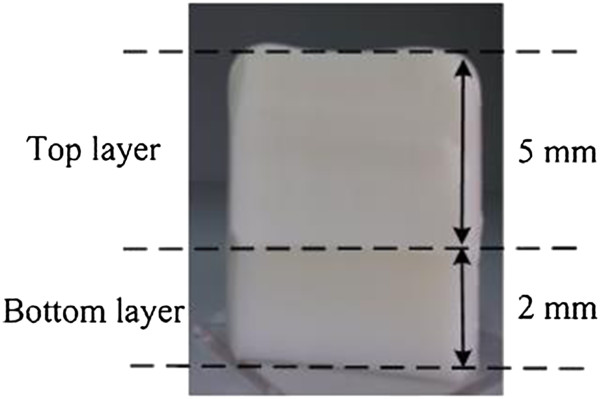
**IL Phantom measurement model.**

A two-layer soft tissue IL phantom was developed, which permitted the probe to go through from the top layer to bottom layer (Figure 3). Solid-solid phantom was utilized in the measurements. The *μ*^*′*^_*s*_ and *μ*_*a*_ values of each layer of the phantoms were measured by our device and validated by ISS Oximeter. Bottom layer: 5 grams of Gelatin power (Sigma, St Louis, MO, USA) made from porcine skin were added into 75 ml boiling water and 25 ml milk (Guangming, China), which was dissolved completely by stirring. Top layer: 5 grams of Gelatin power were added into 20 ml boiling water and 80 ml milk. The bottom layer has *μ*^*′*^_*s*_ = 12 cm^-1^ and the top layer has *μ*^*′*^_*s*_ = 17 cm^-1^. The experimental setup for laboratory phantom measurement was our NIRs measurement device. The depth of the top layer was 5 mm, and that of the bottom layer was 2 mm.

### Pedicle screw insertion experiment

**Figure 4:**
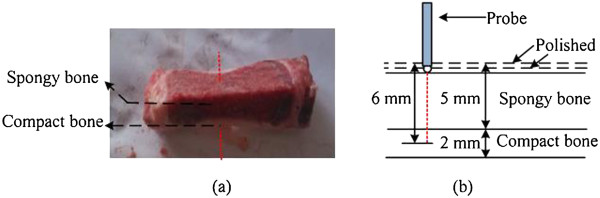
**Detection depth measurement experiment. (a)** the section photograph of vertebra bone: red dotted lines represent measurement lines of the probe; **(b)** experiment diagram: black dotted lines represent polishing lines and red dotted lines represent measurement lines of the probe.

Fresh porcine lumbar vertebrae (n = 10/group), which came from mature porcine cadavers weighting 38–45 kg, were fixed on the bracket. Muscle tissue on bone surface was removed to expose the facet joint. Compact bone and spongy bone were separated from the whole vertebra shown in Figure 4(a). In each dotted line, reduced scattering coefficient (*μ*^*′*^_*s*_) and light spectra were measured, which were used as the important parameters of the NIRs system during the experiment.

Pedicle screw experiment was performed in spongy bone with the depth of 5 mm, which was located upon compact bone within 2 mm depth. The pedicle model including spongy bone and compact bone is shown in Figure 4(b). The fiber probe was placed on the top layer of the pedicle model, and a series data were measured along the red dotted line on the model surface. Specifically, the *μ*^*′*^_*s*_ values and light spectra were recorded every 0.5 s and the data in 10 s (20 values) were averaged. After that, the pedicle model was polished with different thickness. The thickness was 0.2 mm when the probe was 0 to 4 mm away from the top surface and that was 0.1 mm when the probe was 4 to 6 mm away from the top surface. Every time after the surface layer was polished, the measurement steps were repeated till the probe was 6 mm away from the top surface.

## Results

### Monte Carlo simulation of two-layer model

**Figure 5:**
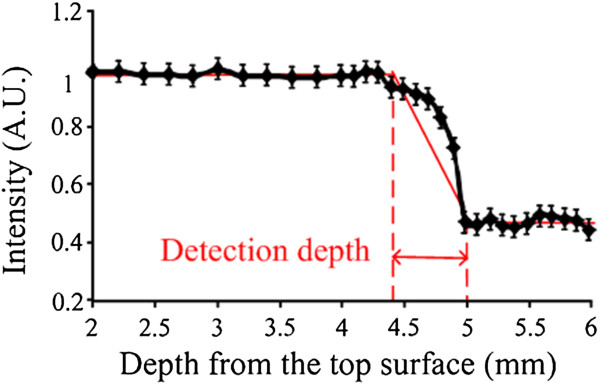
**Monte Carlo simulation results.** Red arrow represents the detection depth.

Figure 5 shows the corresponding simulated reflectance intensity profile of a 2-layer model. As described before, the top layer (*μ*^*′*^_*s*_ = 17.23 cm^-1^ and *μ*_*a*_ = 0.41 cm^-1^) and bottom layer (*μ*^*′*^_*s*_ = 12.02 cm^-1^ and *μ*_*a*_ = 0.21 cm^-1^) were spongy bone and compact bone, respectively. The data were normalized to the reflectance intensity of the first point. The intensity profile begins to decrease slowly at around 4.4 mm, and then remarkably decreases at 5 mm, indicating that the probe passes through the boundary of the two layers and enters the bottom layer. After 5 mm, the data reach a plateau gradually, which suggests the probe is in the bottom layer. The distance between two red dotted lines was defined as detection depth, which is 0.6 mm here. The position of the first red dotted line was set, where the data deviation were larger than 2 times of the standard deviation of the normalized intensity data within the flat region. The last red dotted line was set, where the point was in the border.

### Detection depth measurement of IL Phantoms with a dual-fiber optical probe

**Figure 6:**
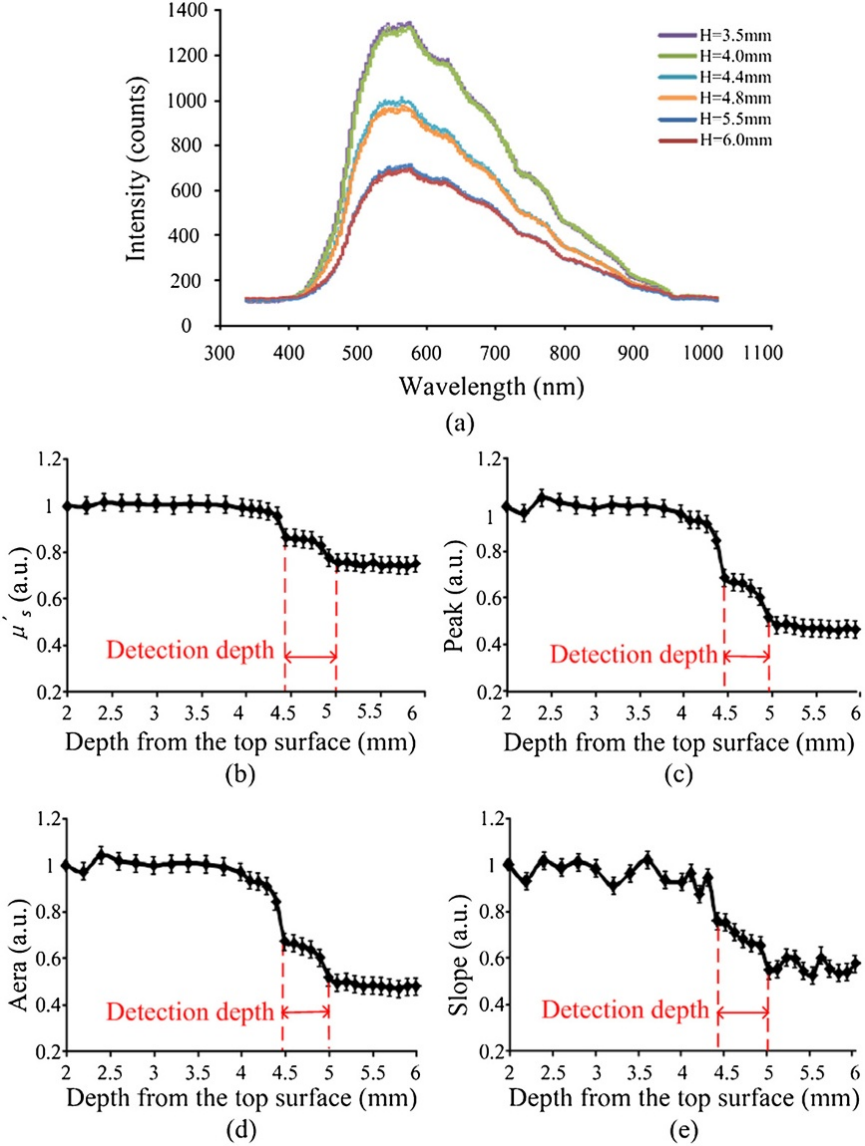
**IL Phantom measurement results. (a)** 6 NIR spectrometers **(b)** the change of the *μ* ^*′*^_*s*_ **(c)** the change of the area **(d)** the change of the peak **(e)** the change of the slope. In Figure 6(a), the dark blue and red lines represent the measurement points in tissue 2. The green, purple, blue and orange lines represent the measurement points in tissue 1.

Figure 6(a) shows the reflectance spectra measured from the top surface to bottom layer, from which the depth is 2–6 mm. The spectra were collected from 340 to 1020 nm shown in Figure 6(a). In normalization, the reflectance intensities were calculated by R = (signal-dark)/ (reference-dark). The dark data were measured when the light source was turned off. The reference data were acquired when the probe was placed on the surface of the standard white sample (Ocean Optics Inc.), which has nearly 99.9% reflection with a flat spectrum. The signal, dark, and reference data were collected with the same integration time of the spectrometer. Figure 6(a) shows 6 reflectance spectra curves, which represent 6 different points along the red line (Figure 4). The curves represent the points in two different tissues, such as tissue 1 and tissue 2. When the points were in the adjacent region near the boundary of tissue 1, the curves were between spongy bones’ curves and compact bones’ curves.

The area values (340–1020 nm), peak values (maximum of spectrum) and slope values (500–550 nm) were calculated from the light spectra and their changes versus the depth from the top surface to bottom are shown in Figure 6(c, d, e). Moreover, we chose the slope’s wavelength at 500–550 nm for the reason that it may have more significant regularity from the spectra in Figure 6(a).

### Detection depth measurement of pig’s pedicle bones with dual-fiber probe

**Figure 7:**
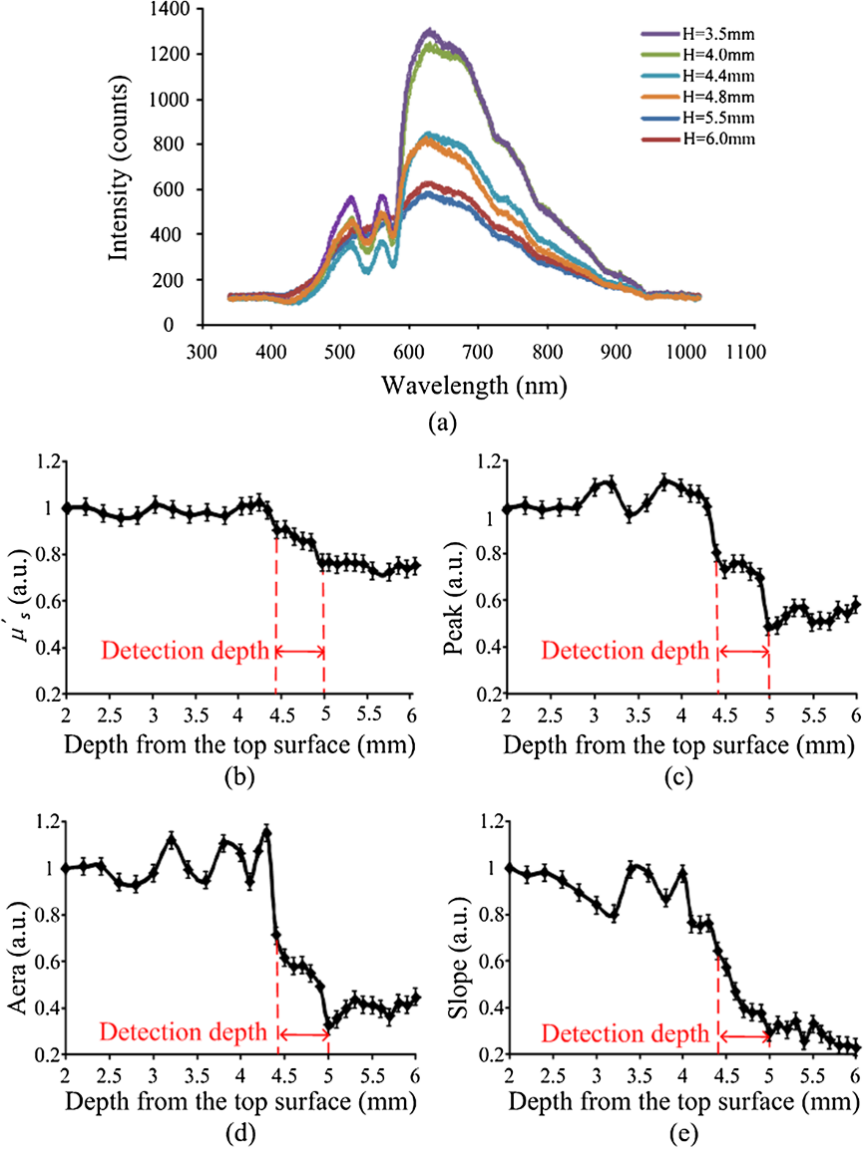
**Experiment results. (a)** 6 NIRs spectrometer: H represents the depth from the top surface **(b)** the change of the *μ* ^*′*^_*s*_ **(c)** the change of the area **(d)** the change of the peak **(e)** the change of the slope. In Figure 5(a), the dark blue and red lines represent the measurement points in compact bone, the green and purple lines represent the measurement points in spongy bone, and the blue and orange lines were just between them, which mean the points in alarm region.

Figure 7 shows the spectra from the pedicle screw experiment (n = 10/group), which is randomly selected data of one group. To determine the values in the experiments, the differences among the values of all points should have a P value <0.05. When the values of pattern factors are the average of tissue 1 and tissue 2, we define the measurement points arriving at alarm regions.

Reflectance spectrum was also collected from 340 to 1020 nm. Figure 7(a) shows 6 spectral curves, which represented 6 points in different tissue locations including spongy bone, compact bones and alarm region. When the points were in alarm region, the curves were just between spongy bones’ curves and compact bones’ curves. The changes versus the depth from the top surface to bottom shown in Figure 7(b, c, d, e) were calculated by using the same algorithm in Figure 6. In Figure 7(b, c, d, e), all the curves show more volatile than that in Figure 6(b, c, d, e).

### Alarm threshold measurement by comparing the experiment results

The results were compared to decide the threshold to alarm during PS insertion surgery by using the NIRs device. In Figure 7(b, c, d, e), the trend of curves was divided into three parts. The pattern factor value was smooth in 0–4.4 mm depth. From depth of 4.4–5 mm, the value remarkably decreased. After the depth of 5 mm, the value was smooth again.

In this paper, the 0.6 mm detection depth indicates the measurement system has a detectable distance from 0 to 0.6 mm. Within the detection depth, the spectra can provide the information of tissues. Therefore, the maximum alarm distance is also 0.6 mm ahead the probe tip.

The different average values are compared and shown in Table 2, which is applied to detect the overlapping degree of average values. Ratio 1 is calculated by Ratio 1 = (average value of spongy bone - average value of alarm region)/average value of spongy bone. Ratio 2 is calculated by Ratio 2 = (average value of alarm region - average value of compact bone)/average value of alarm region. Ratio 1 and Ratio 2 of pattern factor Slope are both the biggest comparing with other pattern factors, and those that of Area are second bigger. So the Slope and Area both can be chosen as the alarm judgment standard to distinguish the location of the probe. Moreover, just considering the average values are not enough for alarm factor in advance. Then, we defined another ratio by spongy bone (minimum value), alarm region (maximum value), alarm region (minimum value) and compact bone (maximum value).

**Table 2:** **Comparing the different average values**

Average value	***μ*** ^*′*^_***s***_(1/cm)	Peak	Area	Slope
Spongy bone	16.91	881.86	185213.61	5.64
Alarm region	14.71	638.5	106896.1	3.07
Compact bone	12.78	463.91	72472.75	1.81
Ratio 1	13.01%	38.11%	42.28%	45.65%
Ratio 2	13.12%	27.34%	32.2%	41.02%

Table 3 shows the pattern factor values of two different tissues and alarm region. Ratio 3 is calculated by ratio 3 = [spongy bone (minimum value) - alarm region (maximum value)]/spongy bone (minimum value). Ratio 4 is calculated by Ratio 4 = [alarm region (minimum value) - compact bone (maximum value)]/alarm region (minimum value). Ratio 3 of Area is biggest and Ratio 4 of Peak is biggest. Combining Ratio 1 and Ratio 2, the Area values could be regarded as the only pattern factor for alarm, because it can be the most clearly distinguished if the probe is in alarm region. Once the Area value of is between 129872 and 89783, it indicates that the measurement point arrives within detection depth and it is time to alarm, which can avoid the cortical breach happening.

**Table 3:** **Comparing the different values**

	***μ*** ^*′*^_***s***_(1/cm)	Peak	Area	Slope
Spongy bone (minimum value)	16.33	825	170300.3	4.87
Alarm region (maximum value)	15.51	649.33	129871.7	4.18
Ratio 3	5.02%	21.29%	23.74%	14.18%
Alarm region (minimum value)	14.6	597	89782.84	2.43
Compact bone (maximum value)	13.12	503.33	80788.38	2.21
Ratio 4	10.14%	15.69%	10.02%	9.21%

## Discussion

In this paper, it was firstly focused on biomedical applications of a small-separation, optical fiber probe to investigate detection depth and identify tissue types by using NIRs. Figure 5 demonstrates the consistent values of the detection depth obtained from the Monte Carlo simulations for this fiber probe, illustrating that the detection depth is 0.6 mm in the simulation. The simulated results provide reference to the next experiments.

In the IL phantom measurements and the bones experiments a decreasing trend of the slope profile was observed when the probe passed through the boundary of two-layer vertebra tissues, especially when the probe was within the detection depth away from the bottom layer surface, shown in Figure 6(b, c, d, e) and Figure 7(b, c, d, e). We defined ratio 1, ratio 2, ratio 3 and ratio 4 for the evaluation of the significance of four pattern factors. If the ratios are higher, the significance of the pattern is better. Considering Tables 2 and 3 the Area pattern factor is most significant in four pattern factors. Comparison with experiment results, the threshold has been defined according to the change of the Area, which would provide an alarm standard to indicate that the screw was not at the correct location during PS placement.

The small-separation probe used in the simulations and experiments has a fiber separation of 200 μm. If the separation is changed, some different results can also be achieved by this method. Moreover, the detection depth for our one NIRs probe depends on the fiber size and probe configuration. In general, the detection depth also depends on wavelength and is relatively constant in the NIRs range [34]. The detection depth will vary largely with wavelength in the wavelength range of 500–650 nm, because of high variation in light absorption due to the existence of haemoglobin in tissue. Further theoretical study is needed if one wishes to prove the answer mathematically.

Former studies have proved that the density of the vertebrae bones was different and the reduced scattering coefficient was related to the density [35, 36]. In this study, we used the porcine vertebrae as the experiment models. Even though the porcine vertebrae are different in terms of optical properties from pig to pig, the results obtained by the method are statistically significant. Thus, we can use the same method to measure the reduced scattering coefficient of the vertebrae bones in the fresh-frozen cadavera experiment in future research.

In addition, several methods for pedicle screw accuracy lack of the real-time ability. Our monitoring technology allows the real-time detection of perforation and the alarm parameter was explored in this paper. The changes of different values in different regions of the trajectory of PS are easy to be achieved and sensitive to distinguish abnormal pedicle screw position. The significant parameter is chosen from four parameters. However, it also needs to assess more accurately and quickly in the clinical setting, so that patients are not exposed to unnecessary risk. Meanwhile, the measured reflectance may cause a different effect in measured data and worsening the spatial resolution because of the scattering nature of light within different tissues. The quantitative detection depth expression in other models will be helpful to interpret the *in vivo* data better, and it will be considered in the future.

The methodology and experimental protocols developed here can also be applied to obtain the detection depth of other optical probes, which are needed to improve the alarm accuracy for better surgery guidance. The knowledge and quantification learned on the detection depth will improve the reliability of this device in PS placement. Moreover, the proposed alarm standard based on detection depth provides a potential for guiding pedicle screw in operation.

## Conclusions

Quantification of the detection depth of a fiber optical probe is important to find an alarm standard to guide surgery. In this work, a two-layer tissue model has been developed to determine the detection depth. Monte Carlo simulations and phantom models were performed to calculate the theoretical detection depth. The porcine vertebral models were also used to obtain optical spectra and reduce scattering coefficient so that the detection depths were deduced. The study shows that a relatively simple value can be associate the detection depth with the scattering property of the vertebra tissue (compact bone and spongy bone) for 200 μm probes, and the result is consistent between the experiment and simulation results. The experiment results based on near-infrared spectroscopy provide a potential for guiding pedicle screw in operation.
